# A novel knock out strategy to enhance recombinant protein expression in *Escherichia coli*

**DOI:** 10.1186/s12934-020-01407-z

**Published:** 2020-07-23

**Authors:** Ashish K. Sharma, Esha Shukla, Deepak S. Janoti, Krishna J. Mukherjee, Joseph Shiloach

**Affiliations:** 1 School of Biotechnology, Jawaharlal Nehru University, New Delhi, India; 2 Biotechnology Core Laboratory, National Institute of Diabetes and Digestive and Kidney Diseases (NIDDK), National Institutes of Health, Bethesda, MD 20892 USA

**Keywords:** *Escherichia coli*, Recombinant protein expression, Signalling control, Cellular stress response, Knock out

## Abstract

**Background:**

The expression of recombinant proteins triggers a stress response which downregulates key metabolic pathway genes leading to a decline in cellular health and feedback inhibition of both growth and protein expression. Instead of individually upregulating these downregulated genes or improving transcription rates by better vector design, an innovative strategy would be to block this stress response thereby ensuring a sustained level of protein expression.

**Results:**

We postulated that the genes which are commonly up-regulated post induction may play the role of signalling messengers in mounting the cellular stress response. We identified those genes which have no known downstream regulatees and created knock outs which were then tested for GFP expression. Many of these knock outs showed significantly higher expression levels which was also sustained for longer periods. The highest product yield (Y_p/x_) was observed in a BW25113Δ*cysJ* knock out (Y_p/x_ 0.57) and BW25113Δ*elaA* (Y_p/x_ 0.49), whereas the Y_p/x_ of the control W3110 strain was 0.08 and BW25113 was 0.16. Double knock out combinations were then created from the ten best performing single knock outs leading to a further enhancement in expression levels. Out of 45 double knock outs created, BW25113Δ*elaA*Δ*yhbC* (Y_p/x_ 0.7) and BW25113Δ*cysJ*Δ*yhbC* (Y_p/x_ 0.64) showed the highest increase in product yield compared to the single gene mutant strains. We confirmed the improved performance of these knock outs by testing and obtaining higher levels of recombinant asparaginase expression, a system better suited for analysing sustained expression since it gets exported to the extracellular medium.

**Conclusion:**

Creating key knock outs to block the CSR and enhance expression is a radically different strategy that can be synergistically combined with traditional methods of improving protein yields thus helping in the design of superior host platforms for protein expression.

## Introduction

The expression of recombinant proteins in *E. coli* triggers a cellular stress response (CSR) which mimics many features of the generalized stress response, the heat shock, the oxidative stress and the stringent response [1–5]. This stress response (CSR) downregulates key metabolic pathways of the central carbon metabolism, ATP synthesis, substrate uptake, ribosomal synthesis as well as activating the glucose overflow mechanism leading to acetate formation [1, 3, 6]. All this leads to a decline in specific growth rates as well as rates of protein expression within a few hours post induction. Any attempt therefore to enhance expression needs to focus on the global cellular machinery to ensure an adequate supply of energy and charged amino acids to the process of protein synthesis. This is especially true for over-expressing systems, where the advent of strong promoters like the T7 or pBAD promoter, has ensured that the bottleneck in protein synthesis is no longer at the transcriptional step. The post induction transcriptomic, proteomic as well as metabolomic profiles of *E. coli* expressing different proteins has been studied in detail to understand the exact nature of the CSR and its effect on protein synthesis rates [2, 7–12]. Strategies to alleviate the deleterious effects of the CSR have been proposed which essentially involve the upregulation of critical genes involved in substrate uptake, ATP synthesis or energy metabolism [13–16]. Earlier studies have shown that several membrane proteins of prokaryotic and eukaryotic origin were expressed at higher levels when the cochaperone (djlA) and the mRNA-degrading inhibitor (rraA) were co-expressed [17]. Other studies have utilized a similar concept and shown that increasing expression of chaperones and folding accessories such as GroEL/ES, DnaK/J-GrpE, Skp, SurA, DegP, DsbC, and FkpA supports improved protein expression [18–22]. Increasing expression of downregulated genes such as prsA and glpF also helped in enhancing the expression levels of IGF-If from 1.8 to 4.3 g/l [23]. These strategies underscore the importance of shifting our focus away from the individual steps of protein synthesis towards a more global approach of identifying key factors in recombinant protein production. However, this methodology is limited to the supplementation of one or two genes, which are assumed to be the most critical in affecting cellular fitness, and hence expression levels. Bioreactor strategies which could help counter this physiological stress and improve cellular health have also been proposed [24, 25].

Instead of trying to ameliorate the undesirable effects of the CSR by individually up regulating key genes, an elegant approach would be to block the mounting of the CSR itself. This would be a truly global approach since blocking the CSR could prevent the down regulation of a large number of genes which synergistically impact on cellular health and consequently on protein production. Given the recent improvements in designing better secretory systems of protein expression in *E. coli* [26–35] we can expect protein concentrations to reach 10 g/l or higher in the extracellular medium if only the expression rates could be sustained for longer time periods. It is important to note that growth would still be adversely affected given the diversion of metabolites towards protein synthesis. One problem with this strategy is the scarcity of information regarding the signalling mechanism which triggers the CSR. Previous studies have identified five signalling pathways the Bae, Cpx, Psp, Rsc, and σ^E^ that sense environmental stress. They can be triggered simultaneously depending on the nature of the stress but each act on different sets of genes to modulate the transcriptomic profile [36–40]. Whether the same signalling pathways would be triggered when the stress is internally generated, as is the case when metabolic fluxes are diverted towards recombinant protein synthesis rather than growth and maintenance, is difficult to answer.

We therefore decided to look at the genes which commonly get up-regulated post induction as potential candidates that signal the mounting of the CSR. Many of these genes had downstream regulatees and knocking out such genes would have a cascade effect on multiple genes, precluding easy analysis. We, therefore, chose to knock out only those genes with no known downstream regulatees in order to understand the effect of the specific gene on the physiological response and its impact on protein expression. It is interesting to note that many of the genes had no direct relationship with the process of protein synthesis and often no known function and yet knocking them out resulted in a significant increase in recombinant protein yields.

## Results and discussion

**Fig. 1:**
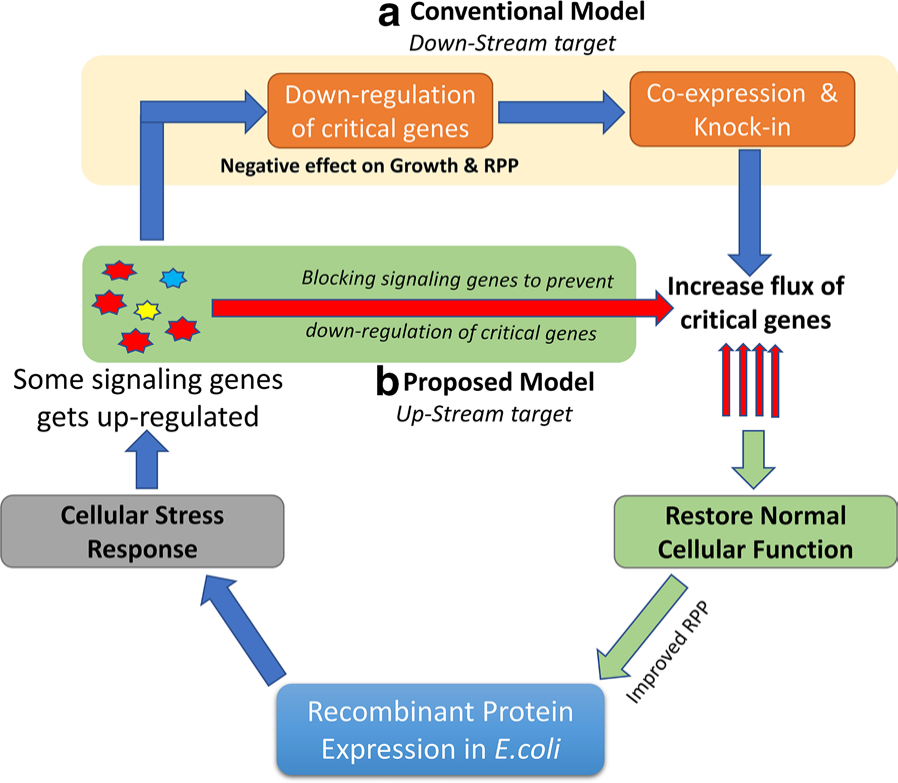
Schematic representation of the conventional and proposed model. **a** Conventional model: emphasis on increasing the expression of down-regulated genes to improve growth and RPP through co-expression or knock-in; **b** Proposed model: targeting the up-regulated genes to block the signaling which initiate CSR

The CSR which is activated in response to the induction of recombinant protein expression can be seen as a feedback inhibition mechanism which operates at the global level to downregulate both growth and protein synthesis. Figure 1 shows a simple schematic of this process where previous studies have tried to address this problem by supplementing the expression of critically down-regulated genes. A problem with this strategy is that it allows us to improve the expression of only a few genes while the CSR downregulates a large number of genes involved in the metabolic fluxes of several pathways. We, therefore, thought of focussing on the upstream part of this feedback loop by attempting to block the signalling messengers that trigger the onset of the CSR. This would be much more effective in disrupting the feedback inhibition loop and hopefully lead to better expression levels.

### Identifying genes responsible for signalling the CSR

**Fig. 2:**
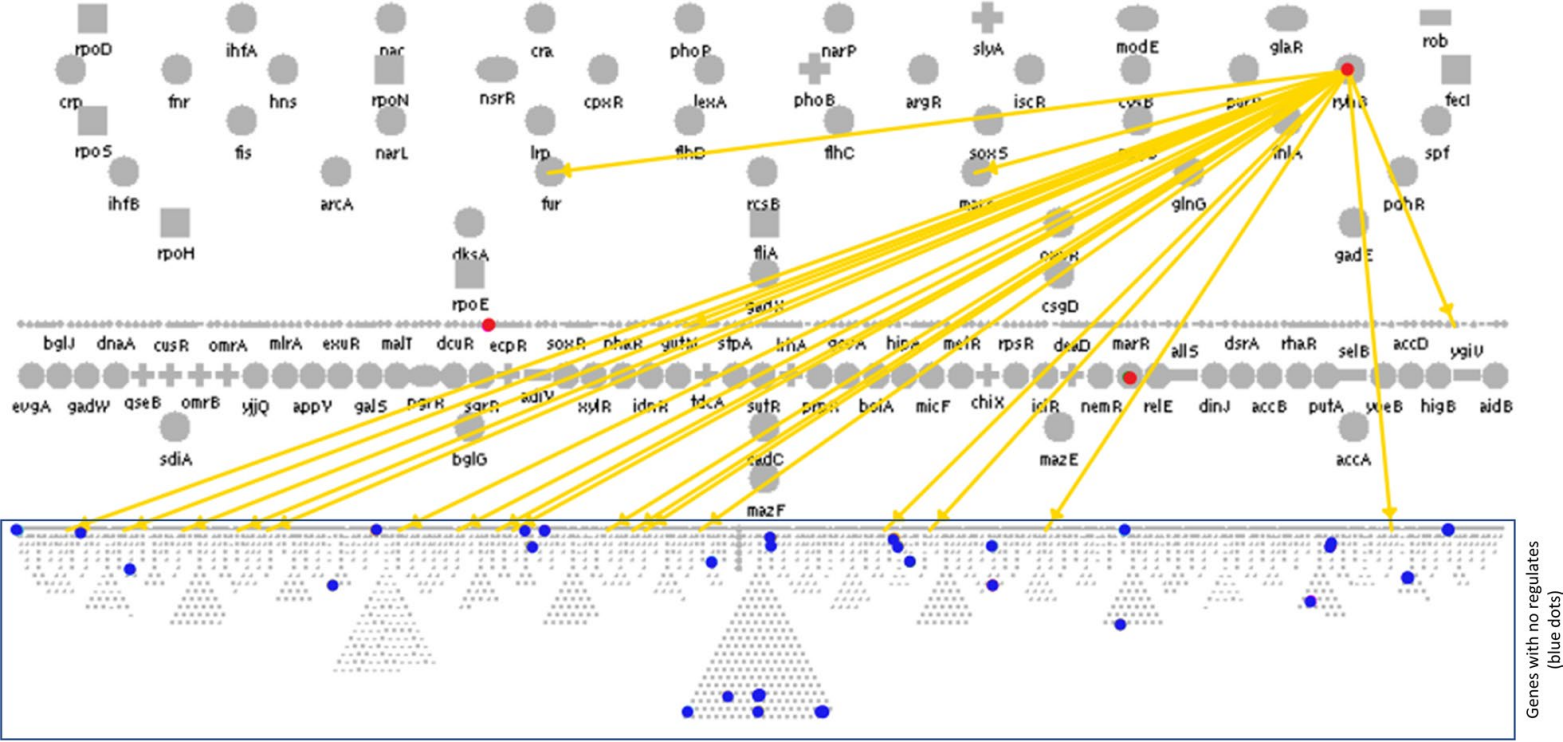
Location of top ten performing genes in the regulatory map of *E. coli* (regulatory overview from EcoCyc) at the bottom layer showing few regulators but no regulatees

Little attention has been paid to the up-regulated genes post induction because their role in recombinant protein production is not clear. We hypothesised that some of these up-regulated genes could be part of the signalling network that activates the CSR. There are some notable differences in the nature of the CSR depending on the type of the expressed protein and whether it accumulates intracellularly (as a soluble protein or inclusion bodies) or gets secreted to the growth medium [6, 41]. In this direction, we previously identified the commonly up-regulated set of genes from transcriptomic studies of cultures expressing different recombinant proteins [6] and these were chosen for further study. These genes were not only up-regulated in the three systems selected but they also remained up-regulated during all time points post induction. We located these genes in the regulatory map of *E. coli* [42, 43] and observed that some of them were higher up in the hierarchical structure. Since these genes control the expression of multiple downstream genes, it would be difficult to identify a specific gene responsible for any observed phenotypic effects. We, therefore, choose, for our knock out studies, only those genes which are at the bottom of the regulatory network and hence have no known downstream regulatees (Fig. 2).

### A model system to study the effect of knock out of these up-regulated genes

In order to explicate the role of these genes, knock outs were created in two different host systems, *E. coli* BW25113 and W3110. This was done to factor in host background effects since small changes in the genome can play a significant role in expression [8, 44, 45] and could also modulate the effect of these knock outs. All these knock-outs as well as the unmodified control cultures were transformed with plasmid pBAD33-GFP (which consisted of the recombinant pBAD33 plasmid having the *gfp* gene under the *ara’* promoter) [6]. A microbioreactor system was chosen to continuously monitor growth and protein expression in a high throughput 48-well format, the pH and dissolved oxygen were also monitored to ensure that cellular health was not compromised by environmental factors.

**Fig. 3:**
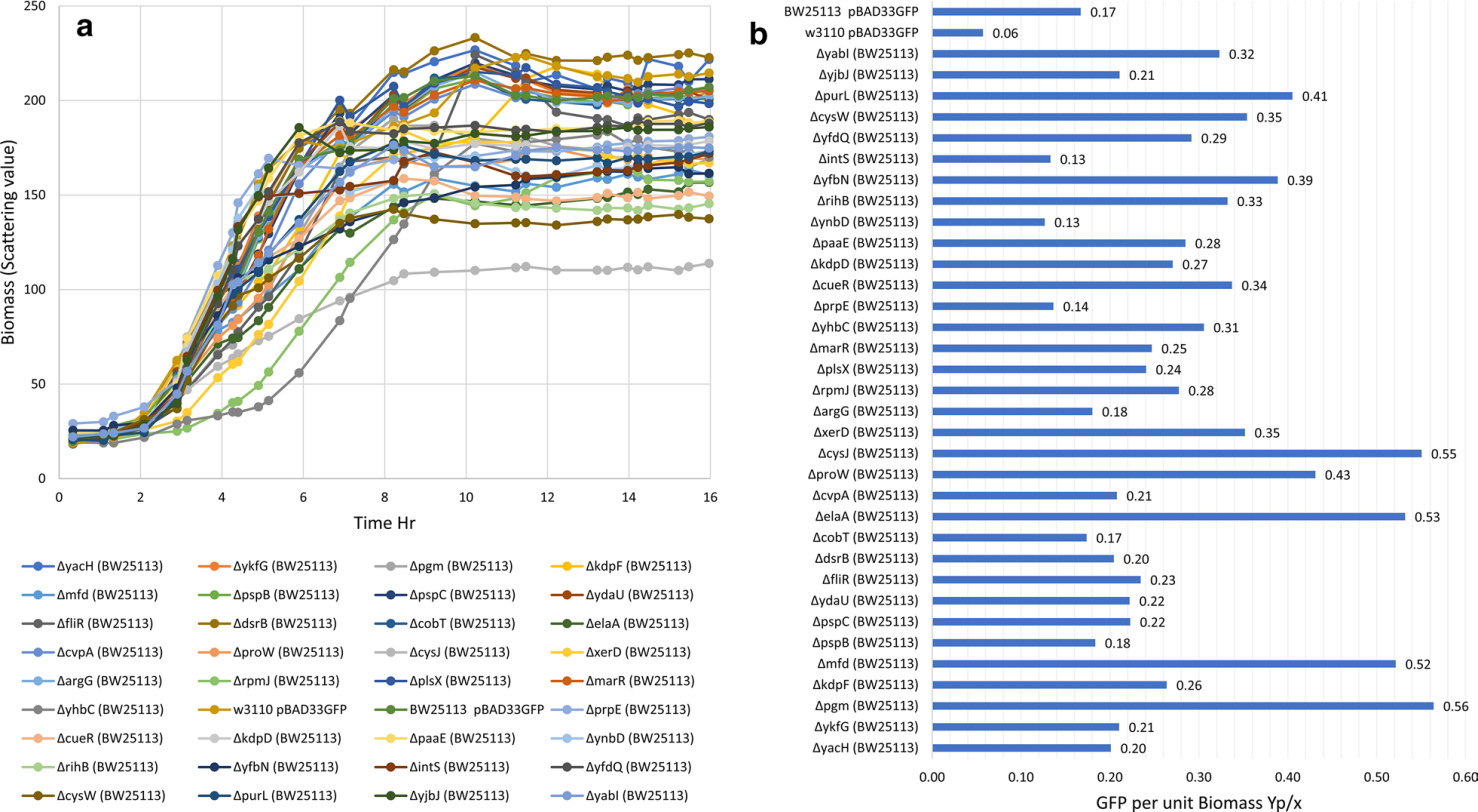
Growth and product kinetics of single gene mutants. **a** Growth profile of single gene mutants, **b** product yield in terms of GFP per unit biomass produced

As seen in Fig. 3b many of the knock outs had a beneficial effect on recombinant protein expression. The control/unmodified strain of W3110 and BW25113 showed a Y_p/x_ of 0.08 and 0.16, respectively. The maximum increase in yield was obtained with BW25113Δ*cysJ*(Y_p/x_ 0.57), BW25113Δ*elaA*(Y_p/x_ 0.49), BW25113Δ*yfbN*(Y_p/x_ 0.42) and BW25113Δ*purL*(Y_p/x_ 0.40). Figure 3a shows the growth profile of these knock outs and it is clear that the growth was not significantly different from the control. Reasonably, the best performing strains often had a slightly poorer growth profile, demonstrating that the large diversion of fluxes towards protein synthesis negatively impacts on growth. Thus, the knock outs which had the highest product concentrations also had high product yields (Y_p/x_) which is a measure of the protein accumulation inside the cell. It is also clear from the expression profile that these knock outs helped in sustaining the product formation rate for significantly longer periods post induction (Additional file 1: Figure S2). As expected, there were some differences in the fold increase of GFP expression obtained in the two host backgrounds with the BW25113 strain giving better results. However, the relative importance of knock outs in providing the highest improvements in performance essentially remained the same. This illustrates that while the host background may affect the fold increase in expression levels, the central role of the knock out in enhancing expression remains unchanged. Table 1 lists the 10 best performing knock-outs in terms of product levels as well as product yields obtained in both host strains of *E. coli.* Additionally, it provides a brief description of the role of these genes from where we observe that many of the top performers have, as of yet, no assigned function and the remaining are distributed between information transfer, carbon utilization, transport, energy metabolism and others. This underscores the fact that the true role of many genes in the dynamic cellular response is yet to be elucidated.

**Table 1:** List of best performing ten single gene knock-outs with their GFP per unit Biomass Y_p/x_, specific growth rate µ (h^−1^) and specific product formation rate q_p(max)_

Gene knock-out	GFP per unit Biomass Y_p/x_	Specific growth rate µ (h^−1^)	Specific product formation rate q_p(max)_
*ΔyacH(W3110)*	0.21	0.36	0.12
*ΔyhbC(BW25113)*	0.32	0.43	0.20
*ΔelaA(BW25113)*	0.49	0.32	0.23
*ΔcysJ(BW25113)*	0.57	0.27	0.25
*ΔmarR(BW25113)*	0.24	0.45	0.15
*ΔyabI(W3110)*	0.32	0.46	0.11
*ΔcysW(BW25113)*	0.34	0.42	0.11
*ΔpurL(BW25113)*	0.40	0.41	0.12
*ΔyfbN(BW25113)*	0.42	0.38	0.08
*ΔcueR(BW25113)*	0.33	0.40	0.10
*W3110*	0.05	0.49	0.08
*BW25113*	0.16	0.47	0.11

### Construction of double knock outs in order to further improve expression levels

**Fig. 4:**
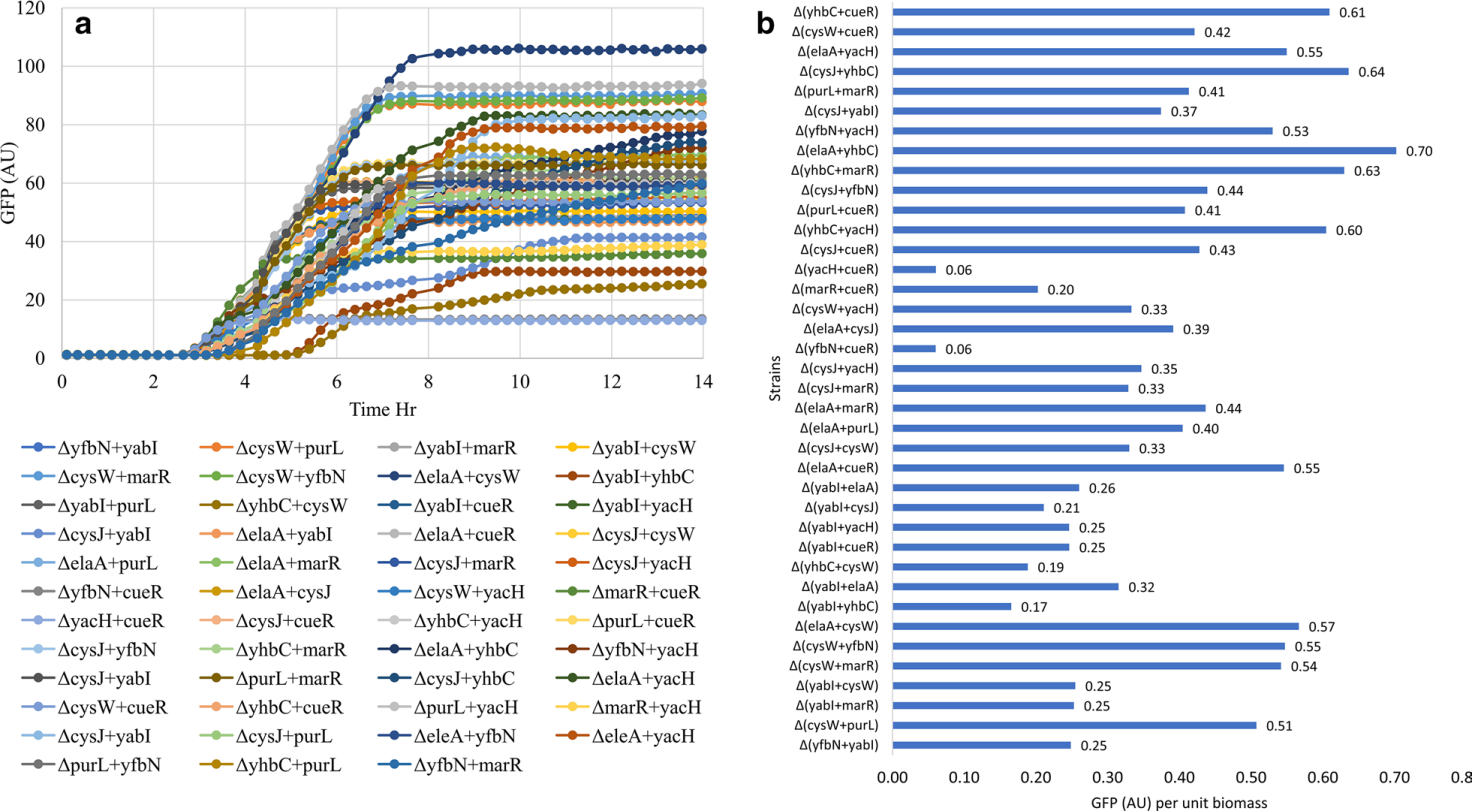
Growth and product kinetics of double gene mutants. **a** Product profile (GFP vs time), **b** product yield in terms of GFP per unit biomass double gene mutant

As stated earlier *E. coli* has multiple signalling pathways which are triggered in various combinations depending on the nature of the environmental stress. Each pathway then acts on a subset of the transcriptome and together they generate the stress response. If this were to be true for the CSR activation by recombinant protein biosynthesis, then different knock outs may well represent the blocking of different signalling pathways. In this case, combinations knock out would be more effective in preventing the downregulation of a larger subset of genes and will contribute to higher level of protein expression. To test this, all possible 45 double knock out combinations were generated from the top 10 single knock outs (Additional file 1: Figure S1). This was necessary since no information was available on whether these knock outs act on the same or different signalling pathways. The expression capability of these double knock outs was tested by transforming them with the pBAD33-GFP plasmid and growing them in microbioreactors with appropriate controls. We observed that some double knock outs performed better and had expression levels higher than the best single knock outs. Figure 4a and b show the expression profile and product yield (Y_p/x_) of these double knock outs. Thus the combination of *∆elaA *+ *∆yhbC, ∆cysJ *+ *∆yhbC, ∆yhbC *+ *∆marR, ∆yhbC *+ *∆cueR, ∆yhbC *+ *∆yacH* and *∆elaA *+ *∆cysW* genes gave Y_p/x_ of 0.70, 0.64, 0.70, 0.61, 0.60 and 0.57 respectively. In comparison, the single gene knock-outs *∆elaA, ∆cueR, ∆marR, ∆yacH, ∆yhbC, ∆cysJ* and *∆cysW* had a lower Y_p/x_ of 0.49, 0.33, 0.24, 0.21, 0.32, 0.56 and 0.34 respectively. Interestingly some double knock outs had a reduced expression level e.g. *∆yfbN *+ *∆cueR, ∆yacH *+ *∆cueR, ∆yabI *+ *∆yhbC, ∆yhbC *+ *∆cysW* and *∆marR *+ *∆cueR* which had Y_p/x_ values of 0.06, 0.06, 0.17, 0.19 and 0.20 respectively, compared to their single gene knock out mutants *∆yfbN, ∆yabI, ∆cueR, ∆marR, ∆yacH, ∆yhbC* and *∆cysW* with Y_p/x_ values of 0.42, 0.32, 0.33, 0.24, 0.21,0.32 and 0.34 respectively, which shows that not all knock out combinations work additively. However, the increase in expression with some double knock outs demonstrates the possible existence of multiple signalling pathways and the need to select proper combinations of knock outs to effectively block the CSR. Table 2 lists the performance of the top ten double knock out combinations and the comparative gains obtained compared with the single knock outs. Growth and product profile comparison of top 10 single and double gene knock outs are provided in Additional file 1: Figure S2.

**Table 2:** Comparative gain (%) in the protein expression in best performing single gene mutants and double gene mutants in comparison to the control strain

Top single gene mutants	Gain % wrt to control (BW25113)	Top double gene mutants	Gain % wrt to control (BW25113)
*∆yacH*	137.53	*∆elaA *+ *∆cysW*	315.01
*∆marR*	147.45	*∆elaA *+ *∆cueR*	279.68
*∆eleA*	239.10	*∆cysW *+ *∆marR*	267.85
*∆cysJ*	185.37	*∆cysW *+ *∆yfbN*	264.64
*∆yhbC*	159.71	*∆cysW *+ *∆purL*	263.82
*∆purL*	210.13	*∆elaA *+ *∆yacH*	247.40
*∆cysW*	268.06	*∆cysJ *+ *∆yabI*	247.33
*∆cueR*	227.85	*∆elaA *+ *∆yhbC*	238.01
*∆yabI*	156.90	*∆eleA *+ *∆yacH*	235.83
*∆yfbN*	258.43	*∆cysJ *+ *∆yhbC*	216.96

### Validation of the improved performance of knock outs by testing for Asparaginase expression

**Fig. 5:**
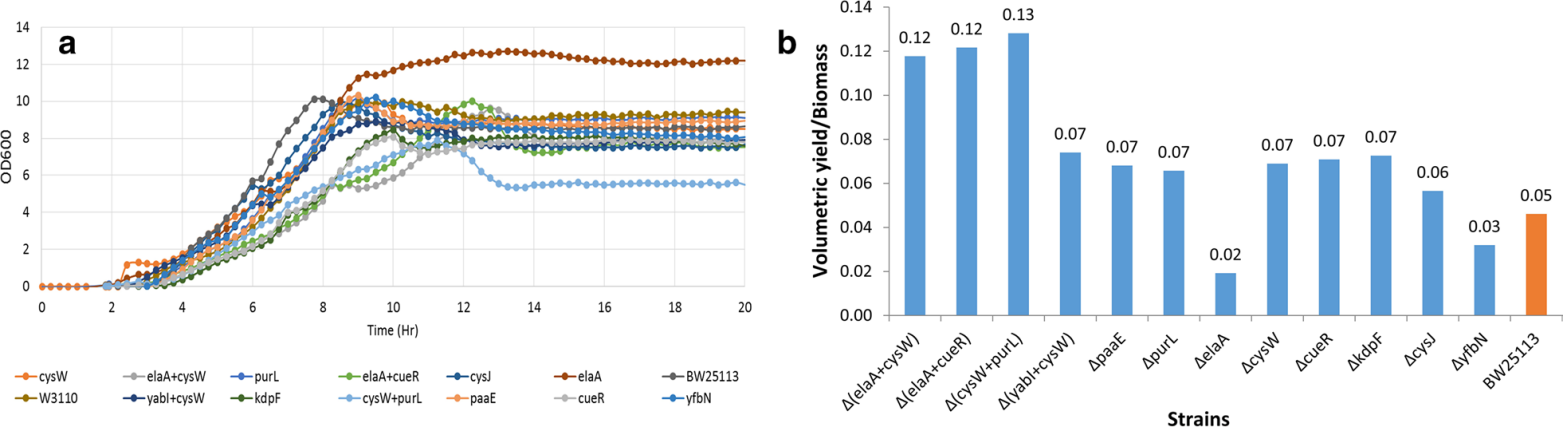
Asparaginase expression profiling. **a** Growth kinetics (OD_600_ vs time), **b** volumetric yield per unit biomass in the control, single gene mutant and double gene mutant

Following the expression of intracellular GFP which may be limited by product build up in the cells, the expression of extracellular asparaginase was tested in the knock outs. Since it gets exported to the extracellular medium it allows for sustained increase in product concentrations, and may therefore be a better system to study the long term expression capability of the host strain. The top four double knock-outs and top six single knock-outs were transformed with the pMAL-p2X plasmid with the asparaginase gene (*ansB*) cloned under the tac promoter [46] and expression was measured 20 h post induction in the extracellular medium. It is important to note that growth ceased within 3–4 h post induction for all the host strains (Fig. 5). However, since asparaginase first accumulates in the periplasm and then gets passively transported it takes time for the protein to accumulate in the extracellular medium. A significant improvement in asparaginase concentrations was observed in both the single knock-out and the double knock-out strains (Table 3) demonstrating the enhanced capability of these knock-outs to sustain expression. The control BW25113 strain produced 30.29 units/ml of asparaginase while the single knock-out mutants *∆purL, ∆elaA, ∆cysW, ∆cueR, ∆cysJ* and *∆yfbN* produced 46.33, 17.82, 44.55, 41.88, 32.97 and 32.08 units/ml asparaginase respectively. Under these conditions the double knock out mutants *∆elaA *+ *∆cysW, ∆elaA *+ *∆cueR, ∆cysW *+ *∆purL* and *∆yabI *+ *∆cysW* showed an activity 70.3, 69.5, 53.46 and 44.55 units/ml asparaginase respectively. Clearly the uniformly high level of increase obtained in these double mutants shows that they are more robust hosts for designing improved expression platforms. The results obtained confirmed our hypothesis that these knockouts (Table 3) are part of different signaling pathways that are primarily responsible for triggering the cellular stress response. It is therefore possible that multiple signaling pathways are activated and these trigger the CSR which is the reason why the double knock outs are more robust and perform better than the single knock outs. The data from both GFP and asparaginase expression showed similar enhancement in yields from the top double knock outs strains. Clearly, the signaling pathways that initiate the onset of the CSR are common to both GFP and asparaginase although there are minor differences in the nature of the CSR that is elicited by different proteins [6, 41]. Also, the top performing double knock outs have significant commonalities in terms of gene combinations emphasizing the point that critical signalling pathways need to be blocked to ease the deleterious effects of the CSR.

**Table 3:** Asparaginase quantitation in supernatant of 20 h post-induction culture of the double knock-out, single knock-outs and control strain

Gene knock-out Strains	Culture OD_600_	Asparaginase Assay (OD_436_)	Asparaginase units/ml	Asparaginase mg/ml
*∆(elaA *+ *cysW)*	7.87	0.7905	70.39	0.35
*∆(elaA *+ *cueR)*	7.52	0.7755	69.50	0.35
*∆(cysW *+ *purL)*	5.49	0.5975	53.46	0.27
*∆(yabI *+ *cysW)*	7.92	0.496	44.55	0.22
*∆purL*	9.10	0.514	46.33	0.23
*∆elaA*	12.19	0.196	17.82	0.09
*∆cysW*	8.50	0.495	44.55	0.22
*∆cueR*	7.78	0.473	41.88	0.21
*∆cysJ*	7.67	0.3745	32.97	0.16
*∆yfbN*	13.20	0.355	32.08	0.16
BW25113	8.65	0.336	30.29	0.15

The results indicate that there is a difference in the profile of the relative advantages of the single knock out mutants between the two expression systems (Fig. 4 and Table 3), since some of the single knock outs that performed well with GFP turned out to be poor performers when expressing asparaginase. However, this difference disappears in the double knock out strains and the best performers are common between both GFP and asparaginase although they are expressed under different promoters. This validates the point that the signaling pathways that activate the CSR are common to all expression systems and shows that the over performance of the robust double knock out system is independent of the host background, the expression system used, and the nature of the protein being expressed. Additionally, since we performed batch culture studies that tend to get nutrient limited in the later part of the cultivation, the results also indicate a better capacity of the knock-out mutants to continue expressing in the late log or stationary phase of the culture.

## Conclusion

The significant improvement expression of both GFP and asparaginase demonstrates the potential of this knock-out strategy to create superior host platforms for recombinant protein production. Since this approach targets a completely different aspect of recombinant protein production viz., blocking the CSR, it should be easy to combine with other traditional methods for enhancing recombinant protein yields.

## Materials and methods

### Strains, plasmids and primers

The *E. coli* strains BW25113 and W3110 were used as the parental/control strain. The plasmids used in this study were (a) pMAL-p2X (AmpR, Ptac promoter expression vector, size 6721 bp) obtained from New England Biolabs, (b) pBAD33-GFP (ChlorR, pBAD promoter expression vector, size 6120 bp) created previously in our lab and (c) pCP20 (ApCm, FLP recombinase expression) sourced from Yale University.

The following primers were used for the confirmation of genetic changes performed in the W3110 and BW25113 strains:

**Table:** 

Name	Sequence (5′ 3′)
*pgmFp*	TTG CAG ACA AAG GAC AAA GC
*pgmRp*	GTG TTT ACG CGT TTT TCA GA
*YacHFp*	TAC CTT TCC AGT CTT CTT GC
*YacHRp*	TGC GAT TTC CTT GAG ATC CG
*elaAFp*	GAT TAC GCC TGA ATT ACC TC
*elaARp*	AGG CAT ACA TCT AAA AGG AG
*cysWFp*	CTC AAT GCT CAT GAT GTT CC
*cysWRp*	CGG CGT GTG GTA GGT CAT TA
*cysJFp*	TCG CTC ATT AGT AGA CAT CT
*cysJRp*	GCT TAC TGG AAC ATA ACG AC
*yabIFp*	ATC CAC CGT GTA TTC ATT GA
*yabIRp*	TAT CTC CTA AAC CCC AAC CA
*marRFp*	CAT TGG GTC GCT TAA TCC AT
*marRRp*	TGT TTA CGG CAG GAC TTT CT
*yhbCFp*	GAC TAT TAA AAG TGG GGA AC
*yhbCRp*	AGA ATA ACT GGG CTT TAG GC
*purL_F*	GAC GAC TTA CCC CAA CTG CT
*purL_R*	GTA CAC CGA AAG CTT AGA AGA
*yfbN_F*	TAT AAC TCG TGT CTG TTA TG
*yfbN_R*	TAT TTA AAG GGG TTT GAC AT
*cueR_F*	GGA GGC GTT GCG CGA ACG AT
*cueR_R*	CAC CCT GCC CGA TGA TGA CA
*k1* (*Kanamycin Fwd Primer*)	CAGTCATAGCCGAATAGCCT
*k2* (*Kanamycin Rev Primer*)	CGGTGCCCTGAATGAACTGC

### Gene knock-out using P1 phage

#### Lysate preparation

An overnight culture of donor strain grown with selection for the marker to be transduced was diluted 1:100 in fresh LB supplemented with 10–25 mM MgCl_2_, 5 mM CaCl_2_, and 0.1–0.2% glucose. This was grown with aeration at 37 °C for 1–2 h. When the cells were in early log phase (slightly turbid, but noticeable growth) 40 µl of P1 phage lysate was added to the culture and continued to be grown at 37 °C. The infected culture was monitored for 1–3 h until the culture had lysed. Once the cells lysed several drops (50–100 μl) of chloroform was added to the lysate and vortexed. This was centrifuged at (3000 rpm, 1–2 min) and the supernatant was transferred to a fresh tube. Again a few drops of chloroform was added and stored at 4 °C.

#### Transduction

The recipient strain was grown overnight in LB medium. On the next day, the cells were harvested by centrifugation (6000 rpm, 2 min) and resuspend in 1/5–1/3 the harvested culture volume in fresh LB + 100 mM MgSO_4_ + 5 mM CaCl_2_. Five reactions were set up by adding recipient bacteria to the tubes with phage and mixed rapidly after addition (a) 100 µl undiluted P1 lysate + 100 µl recipient cells; (b) 10 µl P1 lysate + 100 µl recipient cells; (c) 10 µl P1 lysate + 100 µl recipient cells; (d) 100 µl undiluted P1 lysate + 100 µl LB; and (e) Only 100 µl recipient cells. These five reactions were incubated at 37 °C for 30 min. 200 µl of 1 M Na-Citrate (pH 5.5) and 1 ml LB was added and incubated at 37 °C for 1 h to allow expression of the antibiotic resistance marker. After incubation, cells were spun down at 6000 rpm for 5 min, resuspended in 100 µl LB supplemented with 100 mM Na-Citrate (pH 5.5), vortexed and plated on an appropriate antibiotic-containing plate. Next day the colonies were streaked on a plate spread with the selection antibiotic mixed in 100 µl of 1 M citrate (pH 5.5). Then, a toothpick was used to pick a few colonies and re-streaked on a new plate to get isolated colonies. The re-streaked colonies were checked for the presence of the desired antibiotic cassette by PCR.

#### Removal of antibiotic marker

To remove the antibiotic cassette, the antibiotic-resistant strain was transformed with the FLP recombinase expression plasmid pCP20-Gm, and ampicillin-resistant transformants were selected at 30 °C. After culturing at 37 °C and/or 43 °C, a large majority of transformants lost the FRT flanked antibiotic gene and also the FLP helper plasmid pCP20-Gm and thus, were fully antibiotic susceptible. The resulting strains were tested by PCR using primers flanking the deleted region.

### Batch fermentation

#### Microscale growth and expression profiling

The small-scale batch fermentations in 1.1 ml culture were carried out in a microfluidic BioLector microtiter plate (M2P labs, Germany). The FlowerPlate format of the microtiter plate was used which ensures maximum oxygen transfer rate (OTR). The temperature was set at 37 °C and humidity at 85%. Although the maximum capacity of each well was 1500 µl, experiments were performed in 1100 µl of culture volume and 1400 rpm to maintain highest OTR throughout the run. The pH, DO and OD_600_ was measured online. Each experiment was performed three times with each strain under identical conditions to achieve the biological triplicate for control, single knock-out and double knock-out strains. The biological replicates showed almost identical profiles and for the ease of reporting we mentioned data from one experiment throughout. All the strains were cultured aerobically in commercially available Terrific Broth (TB) medium supplemented with 10 mM MgSO4 and 0.4% glycerol at 37 °C. Recombinant *E. coli* strains carrying pBAD33-eGFP, were induced with 0.2% l-arabinose for eGFP expression while strains harbouring the pMAL-p2X plasmid were induced with 1 mM IPTG. The selection antibiotic used for the plasmids pBAD33-eGFP and pMAL-p2X were chloramphenicol and ampicillin, respectively. The concentrations of antibiotics used were; chloramphenicol 30 µg/ml (1X) and ampicillin 100 µg/ml (1X). In addition, kanamycin 25 µg/ml (1X) was added to maintain the selection pressure on the mutants since the kanamycin resistance gene was used to create the gene deletions. The mutants were grown overnight with shaking (200 rpm orbital shaker) at 37 °C in 3 ml LB tubes. Secondary inoculation was done by adding 1 ml of overnight grown culture in 10 ml of LB medium in 100 ml shake flasks. After ~ 1.5–2.0 h when the OD measured at 600 nm was between 0.8 and 1.5, cultures were induced by adding 1 mM IPTG or 0.2% l-arabinose as per the experimental requirements. After induction, the OD was monitored at 600 nm at regular time intervals and samples were pelleted and stored at − 20 °C, for further analysis.

### Parameter calculations

The biomass was measured using the scattered light with λ_Ex_/λ_Em_ of 620 nm/620 nm. A calibration was performed to derive conversion equation between ‘scattering values’ and OD_600_ which was y = 22.658x + 21.506 with R^2^ = 0.98, where y and x represent scattering value (at gain value 20) and OD_600_ respectively. Y_p/x_ values reported throughout were calculated with the product amount measured in arbitrary units and biomass measured as scattering value. Same setting parameters were used to measure scattering value and GFP fluorescence for each batch run. Therefore the values reported can be used relative comparison among different gene mutants.

### GFP quantitation

In microscale fermentation, GFP was measured online in realtime while culture was growing in the microtitre plates with the excitation and emission filter of 485 nm and 520 nm respectively. Intracellular GFP quantitation performed by collecting 100 µl culture at different time points, OD_600_ was adjusted to 1 with PBS buffer. 1 ml sample was centrifuged at 12,000 rpm for 10 min, the pellet was redissolved in 100 µl PBS buffer and 1X loading buffer. The mixture was boiled for 20 min and centrifuged at 12,000 rpm for 10 min. The whole cell lysate was collected as supernatant and equal volumes loaded on 12% SDS-PAGE gel.

### l-Asparaginase quantitation

l-Asparaginase activity was measured using standard colorimetric assay with Nessler’s reagent (Sigma Aldrich). A diluted enzyme solution and 189 mM l-asparagine to a final volume of 2 ml, adjusted with 50 mM Tris/HC1 buffer, pH 8.6, was incubated at 37 °C  for 30 min. Then 0.1 ml of the appropriately diluted culture supernatant was added to it. The reaction was terminated by adding 0.5 ml of 1.5 M trichloroacetic acid. The solution was centrifuged to remove the precipitated protein at 15,000*g* for 2 min. A 0.2 ml aliquot of the supernatant from the previous step was added to 4.3 ml deionized water and then 0.5 ml ammonia color reagent. This mixture immediately mixed by inversion and after 1 min it was measured spectrophotometrically at OD_436_. The amount of ammonia produced in the reaction mixture was determined from a calibration curve prepared with ammonium chloride following the same procedure.

Genes were classified into different classes using MultiFun classification [47]. Regulatory control including regulatees were analysed using the ‘Regulatory overview’ module of EcoCyc for *E. coli* K-12 [42].

## Supplementary information

**Additional file 1: Figure S1.** Schematic representing the strategy used to create double gene mutants from the screened 10 best performing single gene mutants. **Figure S2.** Growth and product kinetics of 10 best performing (a) single gene mutants and (b) double gene mutants.

## Data Availability

All data generated or analyzed during this study are included in this article and its additional file.
