# Supplementary material for: A novel knock out strategy to enhance recombinant protein expression in Escherichia coli
Source: Microb Cell Fact. 2020 Jul 23;19:148. doi: 10.1186/s12934-020-01407-z (PMC7376861; doi:10.1186/s12934-020-01407-z)

**Additional file**

**A novel knock out strategy to enhance recombinant protein expression in *Escherichia coli***

Ashish K. Sharma^1,2^, Esha Shukla^1^, Deepak S. Janoti^1^, Krishna J. Mukherjee^1^, Joseph Shiloach^2*^

^1^School of Biotechnology, Jawaharlal Nehru University, New Delhi, India

^2^Biotechnology core laboratory, NIDDK, National Institutes of Health, Bethesda, USA

Figure S1. Schematic representing the strategy used to create double gene mutants from the screened 10 best performing single gene mutants.


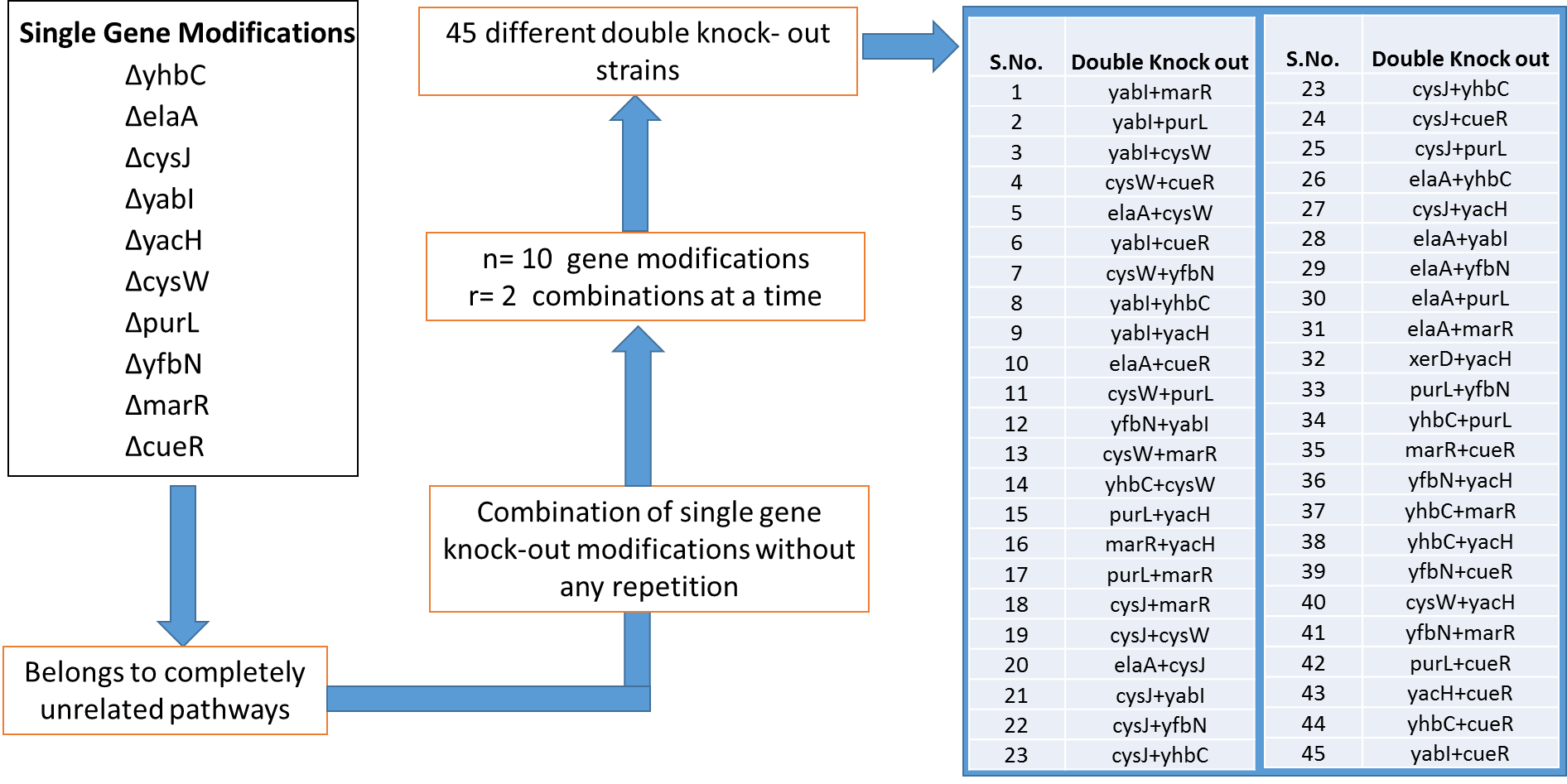


Figure S2. Growth and product kinetics of 10 best performing a) single gene mutants and b) double gene mutants.

a)
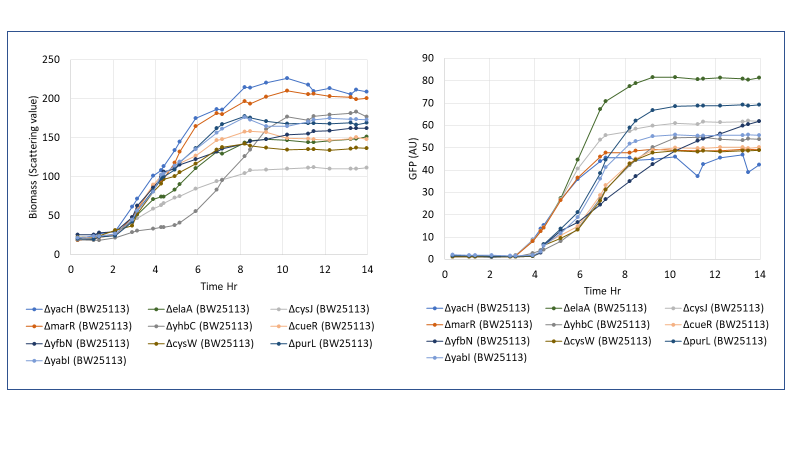


b)


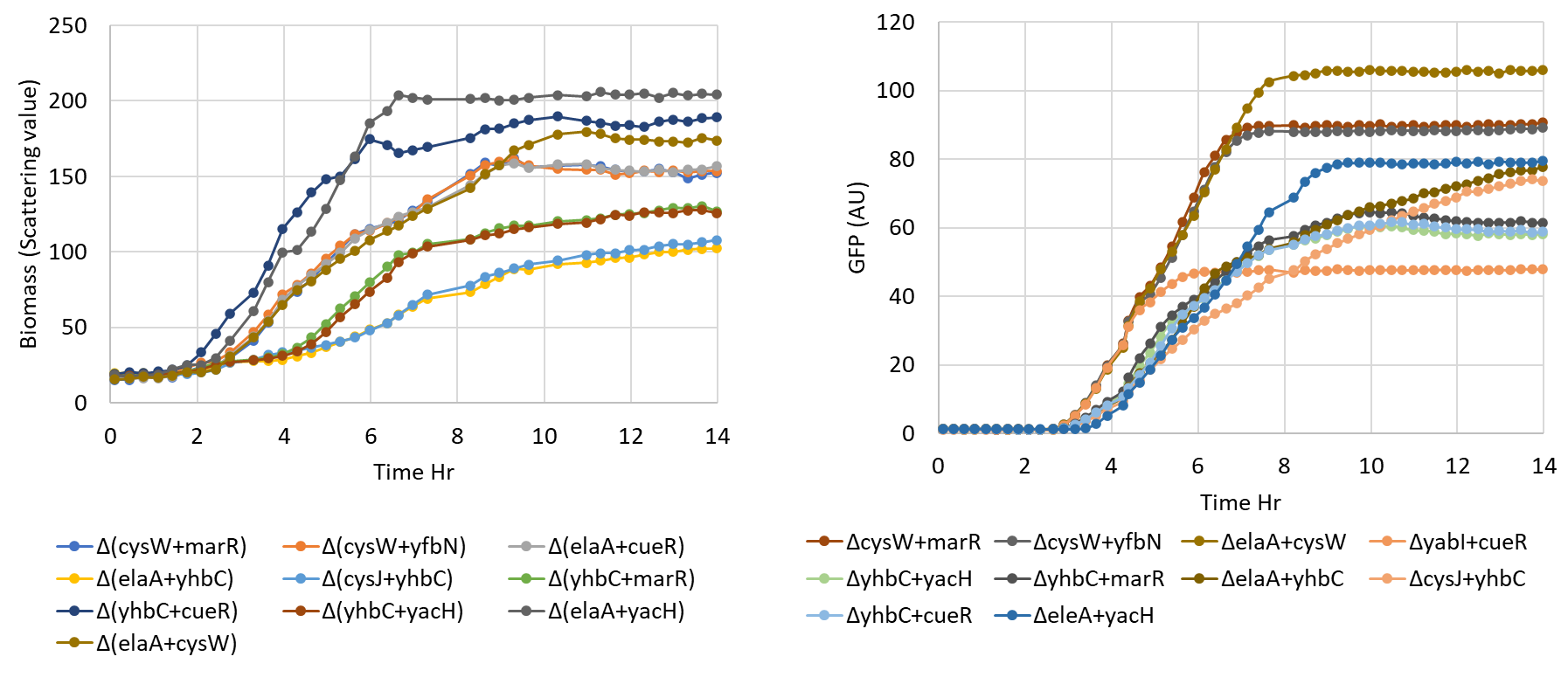

Supplement: Supplementary file 1 — Additional file 1: Figure S1. Schematic representing the strategy used to create double gene mutants from the screened 10 best performing single gene mutants. Figure S2. Growth and product kinetics of 10 best performing (a) single gene mutants and (b) double gene mutants. [file 12934_2020_1407_MOESM1_ESM.docx]
